# Standard setting in Australian medical schools

**DOI:** 10.1186/s12909-018-1190-6

**Published:** 2018-04-23

**Authors:** Helena Ward, Neville Chiavaroli, James Fraser, Kylie Mansfield, Darren Starmer, Laura Surmon, Martin Veysey, Deborah O’Mara

**Affiliations:** 1 Adelaide Medical School, The University of Adelaide, Adelaide, 5005 South Australia; 2 Department of Medical Education, Melbourne Medical School, The University of Melbourne, Melbourne, VIC 3010 Australia; 3 School of Medicine, Griffith University, Parklands Drive, Southport, QLD 4222 Australia; 4 School of Medicine, University of Wollongong, Wollongong, NSW 2522 Australia; 5 School of Medicine, The University of Notre Dame, PO Box 1225, Fremantle, WA 6959 Australia; 6 School of Medicine, Western Sydney University, Penrith, NSW 2751 Australia; 7 Hull York Medical School, York, YO10 5DD UK; 8 Education Office, Sydney Medical School, The University of Sydney, Sydney, NSW 2006 Australia

**Keywords:** Standard setting, Assessment, Preclinical teaching, Medical education

## Abstract

**Background:**

Standard setting of assessment is critical in quality assurance of medical programs. The aims of this study were to identify and compare the impact of methods used to establish the passing standard by the 13 medical schools who participated in the 2014 Australian Medical Schools Assessment Collaboration (AMSAC).

**Methods:**

A survey was conducted to identify the standard setting procedures used by participating schools. Schools standard setting data was collated for the 49 multiple choice items used for benchmarking by AMSAC in 2014. Analyses were conducted for nine schools by their method of standard setting and key characteristics of 28 panel members from four schools.

**Results:**

Substantial differences were identified between AMSAC schools that participated in the study, in both the standard setting methods and how particular techniques were implemented. The correlation between the item standard settings data by school ranged from − 0.116 to 0.632. A trend was identified for panel members to underestimate the difficulty level of hard items and overestimate the difficulty level of easy items for all methods. The median derived cut-score standard across schools was 55% for the 49 benchmarking questions. Although, no significant differences were found according to panel member standard setting experience or clinicians versus scientists, panel members with a high curriculum engagement generally had significantly lower expectations of borderline candidates (*p* = 0.044).

**Conclusion:**

This study used a robust assessment framework to demonstrate that several standard setting techniques are used by Australian medical schools, which in some cases use different techniques for different stages of their program. The implementation of the most common method, the Modified Angoff standard setting approach was found to vary markedly. The method of standard setting used had an impact on the distribution of expected minimally competent student performance by item and overall, with the passing standard varying by up to 10%. This difference can be attributed to the method of standard setting because the ASMSAC items have been shown over time to have consistent performance levels reflecting similar cohort ability. There is a need for more consistency in the method of standard setting used by medical schools in Australia.

## Background

As medical programs are increasingly accountable for the quality of their graduates, the setting of valid and defensible standards is critical. A number of countries, including the USA, Canada and China, have national medical licensing exams to ensure that all medical graduates have achieved a certain standard in knowledge, skills and attitudes required to be a doctor [1–3]. The General Medical Council in the United Kingdom is planning a national medical licensing examination and implementing the examination fully in 2022 [4]. Other countries, such as Australia, are still debating the need for a national approach to setting standards for graduating doctors [5, 6]. In Australia, a national licensing examination has been discussed as a way to overcome the variability in assessment at the various medical schools and also provide a benchmark for new medical schools [5]. Currently in Australia, all medical programs are accredited by the Australian Medical Council, however there is no national exit examination.

One of many challenges posed by a national examination is the setting of a suitable standard for establishing a cut-score or pass mark which allows competent and poor performances to be distinguished [6, 7]. Two major types of standard setting are, norm-referenced (based on a student’s performance relative to the performance of the whole group) and criterion-referenced (referenced to a specified level of performance) [7]. Examples of criterion-referenced standard setting methods include Angoff, Nedlesky and Ebel [8–11]. The use of standard setting panels is also an important consideration and both the number and role of the panel members are important. For instance, a panel may include lecturers, medical scientists and clinicians [12].

In the Angoff method, panel members estimate the proportion of minimally competent students who would respond correctly to each item [8]. The term ‘modified Angoff’ refers to many different variations of the method originally outlined by Angoff, including the use of (Yes/No) decisions rather than proportions [8]. Other variations of modified Angoff include whether item performance data is provided, panel discussions to achieve consensus [13] and whether answers to questions are provided [14–17]. Although there has been previous research on the impact of differences in standard setting methods [18, 19] there have been no previous investigations of standard setting in medical education in Australia.

**Fig. 1:**
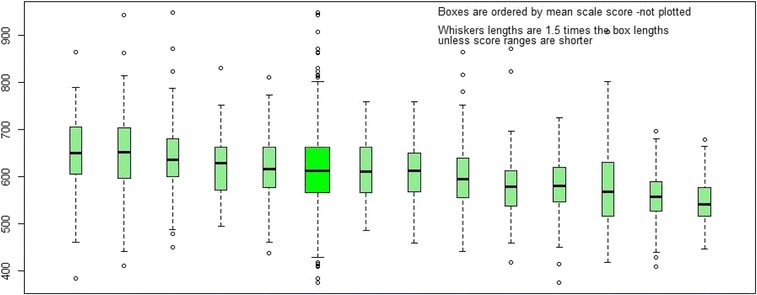
AMSAC medical schools benchmarking performance for 2014*. Note: the box sixth in from the left in bright green is the combination of all schools

The Australian Medical Schools Assessment Collaboration (AMSAC) provides a unique opportunity to conduct an evaluation of standard setting in Australian medical education across similar curricula and student performance levels. AMSAC annually develops a set of 50 benchmarking multiple choice questions (MCQs) in basic and clinical sciences at the pre-clinical level [20]. The level of achievement across AMSAC schools is very similar each year (Fig. 1). In 2014 AMSAC members included 2606 students from 13 of the 19 medical schools in Australia, representing 72% of the 3617 students enrolled in Year 2 of the participating medical programs in that year.

The aims of this study were firstly to identify the methods of standard setting used by Australian medical schools who participated in AMSAC in 2014; secondly to assess the impact of different methods on the school cut-scores for the 2014 AMSAC MCQ items; and thirdly to assess the effects of characteristics of panel members on the standard setting results for each item.

## Methods

This study was conducted collaboratively by assessment representatives from the 2014 AMSAC schools. Approval for the study and the methods used was given by the whole collaboration.

### Standard setting methods used by Australian medical school

A survey was designed to identify the methods of standard setting used in 2014 by the 13 AMSAC schools. A web-based survey was conducted using FormSite software (Vroman Systems, Downers Grove, Illinois, USA) distributed to the AMSAC assessment representative from each school. Participants were asked for details on the method of standard setting used, including several implementation options. (See Appendix for details of the survey and standard setting procedures).

### Standard setting cut-scores

Data for individual panel members who standard set the 2014 AMSAC MCQ items was returned by the assessment representative using a template which requested panel level data as well as the overall result per item and the method of standard setting used. The key characteristics of each panel member were classified by characteristics discussed and agreed upon at the annual 2014 AMSAC meeting; clinician or scientist, experienced or novice standard setter and knowledge of curriculum (extensive, average or low).

### Analysis

Although 12 of the 13 AMSAC schools responded to the survey, only 10 returned standard setting data. Six schools included individual panel member ratings but four schools only returned the overall rating for each item, partly because the method of standard involved a consensus discussion to achieve one rating. One school rated only 20 items and was therefore excluded from the study. The number of panel members for each school was 4, 6, 7, 11, 15 and 25. However, the two schools with the larger panels had collected data over two sessions with different panels and hence not all panel members rated all items. Therefore, school level data were analysed for nine schools, but the analysis of panel members was based only on 28 raters for the four schools with a complete data set.

All data was de-identified before merging, to protect the anonymity of schools and panel members, and in accordance with the ethics requirement for the collaboration. One AMSAC item was excluded from the AMSAC pool due to poor item performance and the data presented here are based on the remaining 49 MCQs. The impact of different standard setting methods on the item ratings and school cut-scores was investigated through descriptive statistics and correlational analyses. The individual panel based data was analysed using the non-parametric statistics Mann-Whitney U Test for the distribution and the Median Test for the cut-score. All analyses were conducted using SPSS Version 24 (IBM. Version 24 ed. Armonk, NY: 2014).

## Results

### Standard setting methods used by Australian medical school

Ten of the 12 AMSAC schools that completed the survey used a criterion referenced method of standard setting, with two using a compromise method; the Cohen and the Hofstee [21, 22]. The most common criterion referenced method of standard setting was a modified version of the Angoff technique in which discussion occurred in an attempt to reach consensus (6 schools). One school reported using the Nedelsky criterion referenced method and one a modified form of the Ebel method. The modified Ebel method used comprised a 3 × 3 grid to classify items by relevance and cognitive complexity, where the original method described by Ebel [10] uses a 4 × 3 classification grid.

The two mixed methods schools reported that they used a different method for different years of their program; Ebel or Modified Angoff and Angoff, Modified Angoff and Nedelsky.

The 10 schools using criterion-referenced standard setting methods varied in the way in which the standard setting was conducted (Table 1). The first half of the table presents variation in the conduct of standard setting. Most schools conducted the standard setting in person, did not have a calibration session (which would build consensus among panellists regarding the characteristics of a minimally competent student), provided the answers to panel members but did not provide prior performance statistics. The majority of schools discussed individual panel member ratings and allowed panel members to adjust their standard setting rating based on the group discussion. Thus, it would appear that some schools used the discussion of ratings to calibrate the standard setting during the process rather than holding a calibration session prior to the standard setting.

**Table 1:** Summary of the differences in the methodologies used by Australian medical schools when undertaking criterion-referenced standard setting

Modifications to standard setting		Modified Angoff	Mixed method	Modified Ebel	Nedelsky	Total
Conduct of the standard setting	Conducted with all panel members in person	4	1	1	1	7
	Conducted electronically	2	1	0	0	3
	Calibration session held	3	0	0	0	3
	No Calibration	3	2	1	1	7
	Answers to question provided to panel members	4	2	0	1	7
	No answers provided	2	0	1	0	3
	Performance data shown to panel member	2	0	0	0	2
	No performance data provided	4	2	1	1	8
	Ratings discussed and changes allowed	2	2	1	1	6
	Consensus decision	2	0	0	0	2
	No discussion or change allowed	2	0	0	0	2
Analysis of standard setting data	Standard set the whole exam	5	2	1	1	9
	Standard set a random selection	1	0	0	0	1
	Used the Mean across all panel members	3	2	1	0	6
	Used the Median across all panel members	1	0	0	1	2
	N/A Consensus used	2	0	0	0	2
	Does not add a measurement error to the cut score	5	2	1	0	8
	Measurement error added to the cut score	1	0	0	1	2
	Standard setting mark used as the cut score	5	2	1	1	9
	Standard setting mark used as a guide only	1	0	0	0	1
TOTAL SCHOOLS		6	2	1	1	10

The second half of Table 1 presents the survey results regarding the use of the standard setting data in determination of assessment cut-score, again reflecting substantial variability. The trend was to standard set the whole exam, use the mean rating across all panel members and items as the cut-score for the minimally competent candidate and to apply this without a measurement error as the passing cut-score on the medical school examination. Variation was also apparent within the group of six schools using some modification of the Angoff.

### School based standard setting cut-scores

**Fig. 2:**
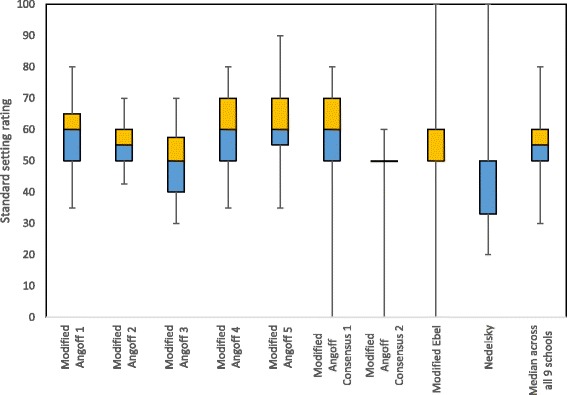
Standard setting item rating by school and method

The median rating and distribution for the nine schools that provided their average or consensus standard setting ratings by item are shown in Fig. 2. The overall median and mean cut-score was 55% with the cut-score for four schools being 50%, one 55% and four 60%. Not all schools have the full distribution of the box plot, because one or both of their quartile scores were the same as the median score; that is Modified Angoff Consensus 2, Modified Ebel and Nedelsky. The last two methods have the widest distributions in part due to the way they are conducted, with scores ranging from 0 to 100 for modified Ebel and 20 to 100 for Nedelsky for these data. The modified Angoff Consensus 2 School gave 39 items a 50% likelihood of being answered correctly by a minimally competent student, regardless of actual difficulty level. The modified Angoff Consensus School 1 has similar overall results to the five schools using the modified Angoff method, except for the longer lower whisker, which reflects the particular conditions of application used within that school (i.e. consensus approach with the full range of percentage scale permitted).

The inter-item agreements between these nine medical schools are presented in Table 2. Although many of the inter-correlations are significant, the inter-correlations are low to moderate (e.g. 0.4 to 0.6). The results indicate that with the same set of items, for a similar curriculum at the pre-clinical stage in a medical program, there is no strong agreement on the level of performance of a minimally competent student across the participating AMSAC medical schools. The results do suggest, however, that there is some agreement between users of the Angoff method, particularly for schools two, four and five.

**Table 2:** Inter-item agreement for each pair of medical school

Medical School	MA 2	MA 3	MA 4	MA 5	Consensus MA 1	Consensus MA 2	Modified Ebel	Nedelsky
MA 1	0.362*	0.221	0.424**	0.465**	0.516**	0.009	0.325*	−0.008
MA 2	1	0.391**	0.549**	0.348*	0.334*	0.064	0.064	0.080
MA 3		1	0.391**	0.371*	0.361*	0.017	−0.116	0.054
MA 4			1	0.632**	0.447**	0.168	0.237	0.346*
MA 5				1	0.352*	0.006	0.352*	0.240
Consensus MA 1					1	−0.001	0.102	0.256
Consensus MA 2						1	0.114	0.119
Modified Ebel							1	0.176
Nedelsky								1

Legend: *MA* modified Angoff

Base: 49 questions for all schools except MA5 where the base is 44 questions

* Correlation is significant at the 0.05 level (2-tailed). ** Correlation is significant at the 0.01 level (2-tailed)

**Fig. 3:**
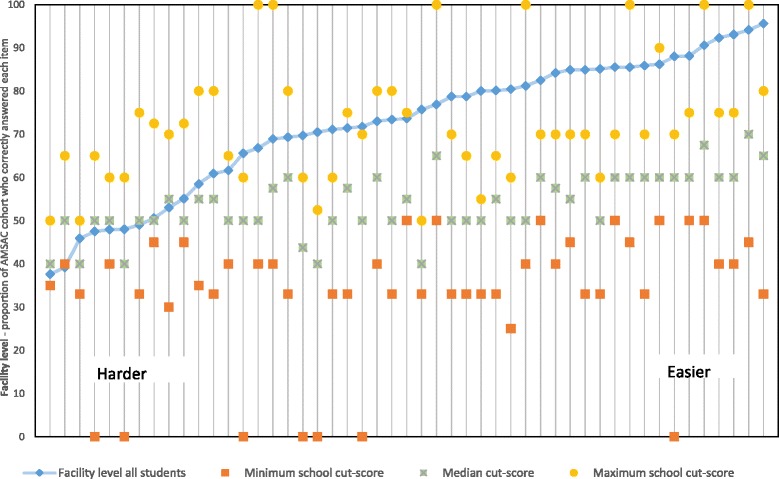
Item facility by standard setting rating summary

Figure 3 shows the range of ratings (median with minimum and maximum) across the nine schools for all 49 items included in the 2014 AMSAC standard setting. The blue series reflects the actual facility level of each item based on the total 2014 AMSAC cohort of 2606 students from 13 AMSAC medical schools (Fig. 1). The items have been ranked from left to right from hardest to easiest. A regression towards the overall median cut-score rating of 55% is evident in the graph, with the maximum, minimum and median dot points trending towards a straight line. This may suggest that panel members did not accurately predict the difficulty level of items, and/or appropriately identify the expected knowledge level of minimally competent students.

### Panel based standard setting cut-scores

The variability in standard setting ratings of the 28 panel members for Modified Angoff schools 1 to 4, reflected that shown in Fig. 3 for the nine schools. The coefficient of variation for the 28 panel members was 13% (mean 55.36, standard deviation 7.32).

The final research question investigated the degree to which item ratings varied by three characteristics of panel members: background of the panel member (scientist or clinician/doctor), level of curriculum engagement (low or average versus high) and experience in standard setting (novice or experienced) for 28 panel members from four of the medical schools that used a modified Angoff approach. There was no significant difference in the distribution of panel members’ standard setting scores across the three characteristics investigated according to the Mann-Whitney U-Test (Table 3). Although the median cut-score of scientists was lower than that for clinicians, the difference was not significant. Panel members with a high knowledge and/or engagement with the curriculum, did however, give significantly lower median cut-scores than those with a low or average knowledge of the curriculum (*p* = 0.044). However, it should be noted that the base for the latter group is low. Novices had a higher median cut-score than experienced Angoff panel members, the difference was not significant according to the median test.

**Table 3:** Statistical results for standard setting panel members characteristics

Panel member characteristics	N	Median	Mann-Whitney U Test		Median test	
			Test value	Significance	Test value	Significance
Background						
Scientist	13	50	112	0.525	0.191	0.718
Clinician/ doctor	15	55				
Total	28	55				
Curriculum knowledge						
Low or Average	8	60	50	0.136	4.725	0.044
High	20	55				
Total	28	55				
Experience with Angoff						
Novice	10	57.5	55	0.099	0.324	0.698
Experienced	18	50				
Total	28	55				

## Discussion

This study addressed three research questions: 1) the methods of standard setting used, 2) the impact of different methods on the school cut-scores for the 2014 AMSAC MCQ items, and 3) the effects of characteristics of panel members on the standard setting results.

The results showed that five methods of standard setting were used in 2014 by AMSAC medical schools. The most common method used was the ‘modified’ version of the Angoff method, which is consistent with recent findings for UK medical education [23] and a recent study by Taylor et al. [24] which revealed differences in passing standards for a common set of items across a number of UK medical schools.

The remaining schools used a range of other standard setting methods including a modified Ebel, Hofstee, Nedelsky and Cohen methods.

Most schools conducted the standard setting process in person with a panel of clinicians and/or scientists, and applied the standard setting process to the whole exam. The cut-scores were usually based on the mean of the panel member ratings, although some schools used the median, and two utilised the SEM to determine the actual final cut-score.

Our study confirms that variation in standards exists between medical schools even when sharing assessment items for similar curricula, at a similar stage of the course. Overall cut-score values ranged from 50 to 60%, with a median of 55% for the 49 AMSAC items used in 2014. The reasons for such variation in the cut-score values for common items may be attributable, in part, to differences in the choice of and/or application of method used for standard setting, although it is also possible that the variation reflects genuine differences in the expectations of students at the various schools. Despite such variations, the differences in student performance on the AMSAC benchmarking questions between medical schools have not been significant over time.

The expected standard of medical students for individual items varied considerably according to the methods used. This was highlighted by the schools that used the Nedelsky and modified Ebel methods, which allowed standards to be set for individual items at the extremes of the available range e.g. from 0 to 100%. A similar factor with the Angoff method is the variation in the lower boundary of judgements. It became apparent in the analyses that some AMSAC schools allowed panel members to use the full percentage range, whilst others chose to set a minimum value (i.e. 20%) reflecting the likelihood of correctly guessing the answer in a five-option MCQ.

Nevertheless, for schools that used a variation of the Angoff approach, many moderately high correlations in individual item cut-scores were found. This suggests that, despite the variations in implementation of the Angoff method, there remains an overall degree of consistency in expected standards.

One of the major findings of this study is the tendency for regression to the mean in panel member judgements. Panel members tended to overestimate performance of minimally competent students on items that were difficult for the whole cohort and underestimate performance on items that were easy for the whole cohort, regardless of panel member background or the method of standard setting used. This is especially true for items with a facility value of 70% or more. Providing performance statistics for questions is recommended by some experts as a way to solve this problem by sharing the prior difficulty level with panel members [24]. However, it is not possible to provide performance data on new questions which may comprise up to one third of a high stakes assessment that needs to be processed in a very short turnaround.

A definition of a minimally competent student, including common characteristics were provided to schools (Appendix), but only three schools conducted the recommended calibration sessions. Our findings point to conceptions of minimal competence which are at odds with actual performance of such students, although whether this is due to a failure to reach adequate consensus about minimal competence, or problems with the application of the standard setting method remains unclear. Ferdous & Plake [25] noted differences in the conceptualisation of ‘minimal competence’ by panel members, particularly between those with the widest variation in expectations of students and our results appear to confirm this. One of the problems in conducting calibration sessions is the difficulty of obtaining time commitment from busy clinician for standard setting workshops.

Future research should investigate the degree to which the facility level for a cohort has a linear relationship with the facility level for minimally competent students. Our preliminary research suggests that the disparity between these two facility levels is greatest for difficult items and smallest for easy items. However, our data are based on different standard setting methods which may account for some of the variations observed.

We found no significant differences in terms of whether panel members were medical clinicians or scientists, or whether they had participated in standard setting previously.

### Limitations

The main limitation of this study is the small number of schools using a similar method of standard setting, which may be affected by variations in implementation of the numerous methods used. Furthermore, there were different response rates to the three parts of this study which might also impact on the clarity of the findings. Twelve of the 13 AMSAC schools participated in the survey, with 10 providing standard setting results at the school level. Although six schools provided individual panel member standard setting data, only data from four schools could be analysed at the panel member level. Furthermore, the number of panel members for these four schools was low (4, 6, 7 and 11) and these results may be less representative than the survey and school based data.

## Conclusions

The results of this study showed that while most participating medical schools utilised the Angoff method to determine the level of minimal competence on benchmarked MCQ items, there was significant variation between schools in the way this approach was implemented. Differences included the use of a calibration session, provision (and timing) of answer keys and/or item analysis to panel members, the degree of panel discussion permitted, and the way the resulting standard was used to set the cutscore. No significant differences in rating behaviour were found according to panel member experience or background, although panel members with a high knowledge of the curriculum had significantly lower expectations of minimally competent students. In general, and in line with previous research, panel members tended to underestimate the difficulty level of hard items and over-estimated the difficulty level of easy items, for all methods and variants, with the median derived standard across schools being 55% for the 49 benchmarking questions.

This study demonstrated that results vary both within and between standard setting methods, and showed that, overall, panel members tend to regress to the mean when trying to predict the difficulty of items for minimally competent students. However, our results also point to a need for more consistency in the method and implementation of standard setting used by medical schools in Australia, especially those which elect to share items, since with the current variability in standard setting approaches and implementation, it is not possible to determine whether the observed variation in cut-score values for common items is due to the variations in standard setting, or genuine differences in the expectations of minimal competence at the various schools. Future research based on consistent implementation of a common standard setting method is therefore crucial to better understand the key factors involved in achieving comparable standard setting results across schools, and in order to facilitate meaningful benchmarking outcomes; an extension to this study has already seen several medical schools implementing an agreed standard setting method and applying that to a subsequent implementation of AMSAC items. Finally, this study exemplifies how a collaboration such as AMSAC, which currently includes 17 of the 19 Australian medical schools, can provide an invaluable opportunity to share expertise and conduct methodological research to improve assessment practices.

## Appendix

### Survey and standard setting procedures

AMSAC medical schools currently use a range of standard setting techniques including but not limited to Angoff, Nedelsky and Cohen.

It was agreed that each school would use their preferred method of standard setting for as many of the 2014 AMSAC questions as possible and return a summary of the method and the results for collation.

Schools summarised the following:

- The method used
- The number of assessors
- The calibration session, if applicable
- Individual panel member data as well as an overall figure, if possible

The purpose of standard setting is to differentiate between students who can demonstrate sufficient knowledge to be safe and competent practitioners and those who cannot. Some methods of standard setting use the term *borderline students*, which can be misinterpreted as students you are unsure about and need to re-assess. We recommend using the term, “the Minimal Competent Student”, i.e. what a medical student who is just acceptable should know or be able to do.

A minimally competent medical student is one who meets the standard, though barely, at the stage the AMSAC questions are used. A minimally competent student has enough of the requisite knowledge and skill to do the job, i.e. they are borderline, but acceptable.

The Angoff method defines the cut-off score as the lowest score the **minimally competent** student is likely to achieve. Students scoring below this level are believed to lack sufficient knowledge or skills to safely progress.

In performing an Angoff Review, each subject matter expert is asked to assign a number to each test item, based on the percentage of minimally competent students who might reasonably be expected to answer it correctly. One way to present this to the expert panel is to ask them to imagine they have 100 minimally competent students sitting in a room and ask them how many they think would get the question right. With traditional five-choice items, the lowest number assigned would be 20, which represents the percentage of students who might be expected to get the item correct by guessing.

Angoff ratings by subject matter experts are pooled and averaged to yield an overall average for the test. This overall average is the expected total score for a minimally competent student and becomes the tentative passing point, expressed as a percentage of possible total point.

## Abbreviations

AMSAC: Australian Medical Schools Assessment Collaboration
MCQ: Multiple choice question
